# Supplementary material for: Anomalous behavior of membrane fluidity caused by copper-copper bond coupled phospholipids
Source: Sci Rep. 2018 Sep 20;8:14093. doi: 10.1038/s41598-018-32322-4 (PMC6148289; doi:10.1038/s41598-018-32322-4)
Supplement: Supplementary file 1 — Supplementary Information [file 41598_2018_32322_MOESM1_ESM.doc]

**Supplementary Information**

**Anomalous behavior of membrane fluidity caused by copper-copper bond coupled phospholipids**

Xiankai Jiang^1†^, Jinjin Zhang^2†^, Bo Zhou^3†^, Pei Li^4^, Xiaojuan Hu^2^, Zhi Zhu^5^, Yanwen Tan^4^, Chao Chang^6*^, Junhong Lü^2*^, Bo Song^5*^

^1^School of Mathematical Sciences and Chemical Engineering, Changzhou Institute of Technology, Changzhou 213032, China

^2^Division of Physical Biology and CAS Key Laboratory of Interfacial Physics and Technology, Shanghai Institute of Applied Physics, Chinese Academy of Science, Shanghai 201800, China

^3^School of Electronic Engineering, Chengdu Technological University, Chengdu 611730, China

^4^State Key Laboratory of Surface Physics and Department of Physics, Fudan University, Shanghai 200433, China

^5^Terahertz Technology Innovation Research Institute, Shanghai Key Lab of Modern Optical System, Terahertz Science Cooperative Innovation Center, School of Optical-Electrical Computer Engineering, University of Shanghai for Science and Technology, Shanghai 200093, China

^6^Key Laboratory for Physical Electronics and Devices of the Ministry of Education, Xi'an Jiaotong University, Xi’an 710049, China

**Contents**

**S1: Bond lengths of the M-O and M-M bindings in States I, I’ and II**

**S2: Bond orders for the bonds M-O and M-M in States I, I’ and II**

**S3: Further analyses of Cu-Cu and Cu-O binding characteristics**

**S4: Improved force fields of Cu related bonds based on the DFT calculations**

**S5: Comparison between PC and PG of adsorbing Cu^2+^ ion**

**S6: Fluidity of DMPC/DMPG systems in the presence of various metal ions**

**S7: FRAP experiments on the supported lipid bilayers prepared from DOPG, DOPC and tail-labeled NBD-PE.**

**S1. Bond lengths of the M-O and M-M bindings in States I, I’ and II**


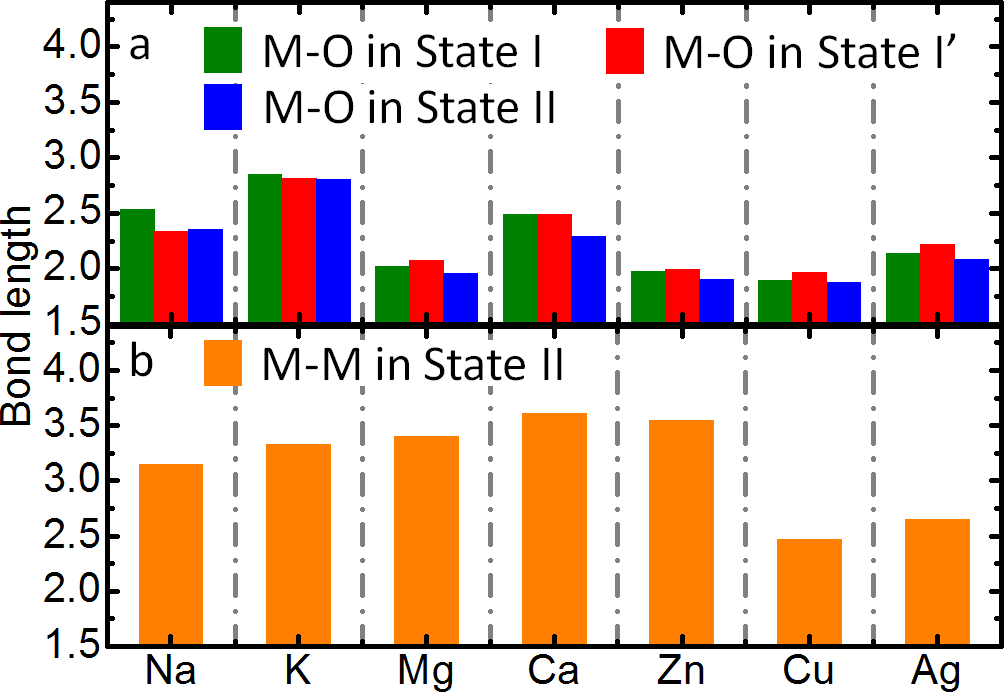


**Supplementary Figure S1. Bond length of the M-O and M-M binding in States I, I’ and II with M = Na^+^, K^+^, Mg^+^, Ca^2+^, Zn^2+^, Cu^2+^ and Ag^2+^. (a)** M-O in States I, I’ and II. The averaged value is applied over M-O2 and M-O3 for State I’, and over M-O1, M-O2, M-O3 and M-O4 for State II. **(b)** M-M in State II. The Cu-Cu and Ag-Ag distances are obviously less than others in State II.

**S2. Bond orders for the bonds M-O and M-M in States I, I’ and II.**

**Supplementary Table S1. Calculated bond order (B.O.) for the bonds M-O and M-M in States I, I’ and II.** Cu-Cu and Ag-Ag bond orders were clearly larger than the others in State II.

| State | Bond | Na | K | Mg | Ca | Zn | Cu | Ag |
| --- | --- | --- | --- | --- | --- | --- | --- | --- |
| State I | M-O1 | 0.050 | 0.030 | 0.177 | 0.095 | 0.267 | 0.355 | 0.270 |
| State I’ | M-O1 | 0.002 | 0.026 | 0.136 | 0.087 | 0.013 | 0.095 | 0.015 |
|  | M-O2 | 0.047 | 0.024 | 0.128 | 0.084 | 0.218 | 0.297 | 0.219 |
|  | M-O3 | 0.001 | 0.030 | 0.107 | 0.097 | 0.246 | 0.295 | 0.207 |
|  | M-O4 | 0.062 | 0.025 | 0.134 | 0.068 | 0.014 | 0.005 | 0.033 |
| State II | M1-O1 | 0.059 | 0.034 | 0.177 | 0.117 | 0.245 | 0.337 | 0.273 |
|  | M1-O2 | 0.043 | 0.041 | 0.170 | 0.113 | 0.239 | 0.349 | 0.248 |
|  | M2-O3 | 0.071 | 0.064 | 0.143 | 0.116 | 0.223 | 0.358 | 0.270 |
|  | M2-O4 | 0.049 | 0.046 | 0.144 | 0.116 | 0.200 | 0.349 | 0.268 |
|  | M1-M2 | 0.026 | 0.082 | 0.012 | 0.024 | 0.010 | 0.420 | 0.298 |

**S3. Further analyses of Cu-Cu and Cu-O binding characteristics**

Apart from identifying bond critical points (BCPs), “atoms in molecules” (AIM) theory[^1^](#_ENREF_1) gives very useful information about the nature of bonding in interacting systems. More recently, the researchers proposed the judging criteria of the nature of chemical bond with the electron densitie *ρ*, Laplacian ∇^2^*ρ*, and the ratio |*V*_C_|/*G*_C_ of the potential-energy density *V*_c_ and kinetic-energy density *G*_c_ at the critical point.[^2-4^](#_ENREF_2) When |*V*_C_|/*G*_C_ < 1, the binding of the atoms is an ionic interaction. When |*V*_C_|/*G*_C_ > 2, the binding is a covalent interaction. When 1 < |*V*_C_|/*G*_C_ < 2, it is the binding with intermediate character between ionic and covalent interactions. Later, Shrabani *et al* investigated the nature of Cu-Cu bond as well as the binding of copper ions and their coordinate atoms in dinuclear copper(I) complexes with the judging criteria. They found that these interactions had significant covalency.[^5^](#_ENREF_5) Here, the electron densities *ρ*, Laplacians ∇^2^*ρ* and the ratios |*V*_C_|/*G*_C_ at the identified BCPs of Cu-Cu and Cu-O chemical bonds were analyzed. For the Cu-Cu and Cu-O bindings, the values of *ρ*, ∇^2^*ρ* and |*V*_C_|/*G*_C_ are tabulated in Table S2. At the BCP point along the Cu-Cu vector, we found small positive values of electron density *ρ* (0.043) and its Laplacian ∇^2^*ρ* (0.053) value, which indicate there are coordination electrons obviously localized between copper atoms and the depletion of charge density between the nuclei. Moreover, the |*V*_C_|/*G*_C_ (1.487) ratio fall in the range 1 < |*V*_C_|/*G*_C_ < 2, which suggests that Cu-Cu bond has some significant covalency. Additionally, the values of *ρ*, positively ∇^2^*ρ*, and |*V*_C_|/*G*_C_ are 0.114 eÅ^-3^, approximately 0.72 eÅ^-5^, and approximately 1.06 at the BCPs between the copper and [oxygen](javascript:void(0);) atoms, respectively. Therefore, Cu-O binding is also an interaction that is not purely ionic but partly covalent.

**Supplementary Table S2. Atoms-in-molecules (AIM) and electron localized function (ELF) analyses of Cu-Cu and Cu-O bindings. The labels *ρ* [eÅ^-3^] and ^2^*ρ* [eÅ^-5^], represent the electron density and its Laplacian in AIM analysis. |*V*_C_|/*G*_C_ is applied for further determining the characteristic of the binding.**

| BCP | *ρ* | ∇^2^*ρ* | |*V*_C_|/*G*_C_ | ELF |
| --- | --- | --- | --- | --- |
| Cu1-Cu2 (3, -1) | 0.043 | 0.053 | 1.487 | 0.254 |
| Cu1-O1 (3, -1) | 0.114 | 0.721 | 1.063 | 0.139 |
| Cu1-O2 (3, -1) | 0.114 | 0.717 | 1.064 | 0.139 |
| Cu2-O3 (3, -1) | 0.114 | 0.717 | 1.061 | 0.139 |
| Cu2-O4 (3, -1) | 0.114 | 0.721 | 1.059 | 0.139 |

**S4. Improved force fields of Cu related bonds based on the DFT calculations**

In order to prepare the force field for our molecular dynamics simulations (MD), the charges of atoms in the headgroup of phospholipid were calculated. NBO charge analysis was utilized for the atoms. Charge transfer situation was determined by comparing NBO charge of the phosphate group before and after the PL-*di*Cu-PL structure formation. After that, following similar protocols used in our previous efforts of force field development,^6^ we improved the atomic charges for each atom in the phosphate group of the phospholipid according to the proportion of the charge transfer. The results for PL-*di*Cu-PL structure together with the corresponding atomic ID are shown in Table S3. The Lennard-Jones potential of copper atom in the AMBER force field was used, with a radius of cross-section *σ*_Cu_ = 3.4 × 10^-1^ nm and a depth of the potential well *ε*_Cu_ = 3.6 × 10^-1^ kJ·mol^-1^.

The calculated bond order (B.O.) and AIM analyses of the M-O and M-M bindings in PL-*di*Cu-PL structure showed that Cu-O and Cu-Cu bonds had partly covalent character (see the details in the supplementary Sect. 1). Therefore, during the MD simulations, the head groups of two adjacent lipids were linked by the Cu-O (0.1906 nm) and Cu-Cu (0.2584 nm) bonds with the angle O-Cu-O (172.53°) according to the DFT-optimized structure. The harmonic potential force constants of Cu-O, Cu-Cu stretching and the bond-angle O-Cu-O vibration were also obtained from the DFT calculations, which were 483660 kJ mol^-1^nm^-2^, 135450 kJ mol^-1^nm^-2^ and 800 kJ mol^-1^rad^-2^, respectively.

**Supplementary Table S3. Improved force fields specifically for Cu-Cu and Cu-O bindings in PL-*di*Cu-PL structure based on the natural-bond-orbital (NBO) analysis.** The charges are given in the unit of *e* = 1.60219 × 10^−19^ C. The charges of other atoms in the phosphate group of phospholipid are directly employed from the CHARMM36 force field.

| PL (CHARMM36) | | | | PL-*di*Cu-PL (NBO fit) | | | |
| --- | --- | --- | --- | --- | --- | --- | --- |
| No. | Atom type | Atom name | Charge | No. | Atom type | Atom  name | Charge |
| 1 | PL | P | 1.500 | 1 | PL | P | 1.750 |
| 2 | O2L | O13 | -0.780 | 2 | O2L | O13 | -0.550 |
| 3 | O2L | O14 | -0.780 | 3 | O2L | O14 | -0.550 |
| 4 | OSLP | O12 | -0.570 | 4 | OSLP | O12 | -0.410 |
| 5 | OSLP | O11 | -0.570 | 5 | OSLP | O11 | -0.410 |
|  |  |  |  | 6 | CU | CU1 | 0.970 |
|  |  |  |  | 7 | CU | CU2 | 0.970 |

**S5. Comparison between PC and PG of adsorbing Cu^2+^ ion**

Our MD simulations suggest that Cu^2+^ ions much prefer staying around the PG region of membrane to the PC region. We performed simulations of CuCl_2_ with PC and PG bilayers, respectively, in presence of water. The radial distribution function of Cu^2+^ to the carboxyl oxygen atoms of lipids in the bilayer was calculated (Supplementary Fig. S2). One peak was clearly observed at approximately 0.25 nm for both cases, meaning that there are Cu^2+^ ions close to the surfaces of PC and PG bilayers. The peak height for PG bilayer was three times of that for PC bilayer, indicating that the probability of Cu^2+^ ions close to PG region is significantly higher than that close to PC region. This can be attributed to the repulsion of Cu^2+^ with the head group [N(CH_3_)_3_]^+^ of PC (Supplementary Fig. S2 middle). Therefore, in our *ab initio* calculations and MD simulations, we just investigated the interaction of Cu ions with PG lipids.


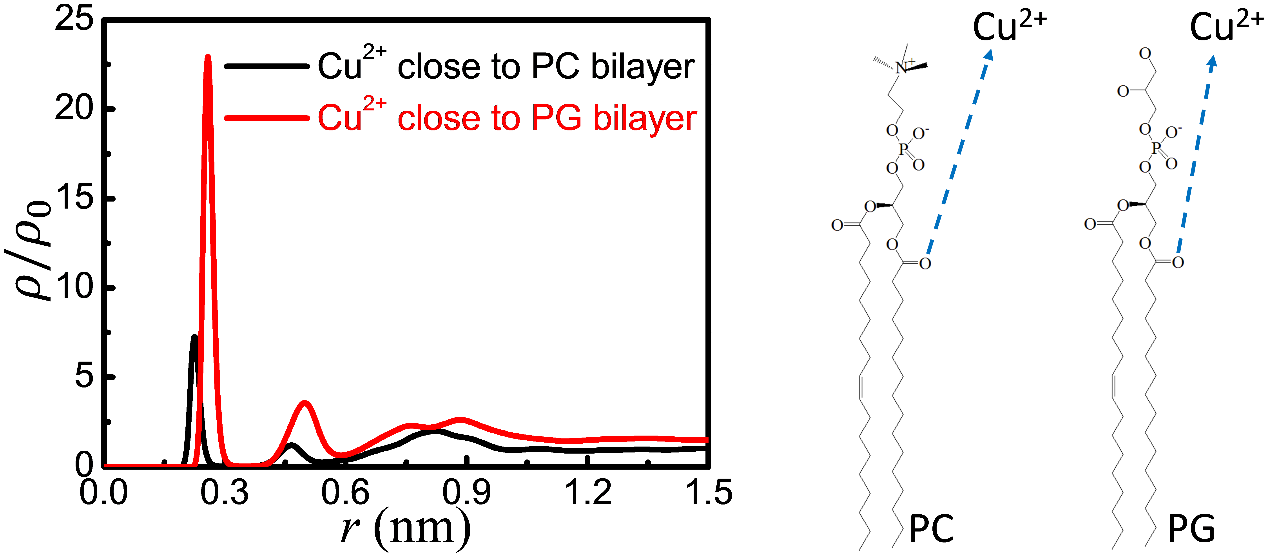


**Supplementary Figure S2. Radial distribution function of Cu^2+^ ions relative to the carboxyl oxygen atoms of phospholipids in the PC (black) and PG (red) bilayers.** The functions are calculated based on MD results. The labels *ρ* and *ρ*_0_ indicate the density of Cu^2+^ and that in bulk water, respectively.

**S6. Fluidity of DMPC/DMPG systems in the presence of various metal ions**

We have performed the fluidity experiments both with DMPC/DMPG (Supplementary Fig. S3), and obtained the similar results of DOPC/DOPG systems.


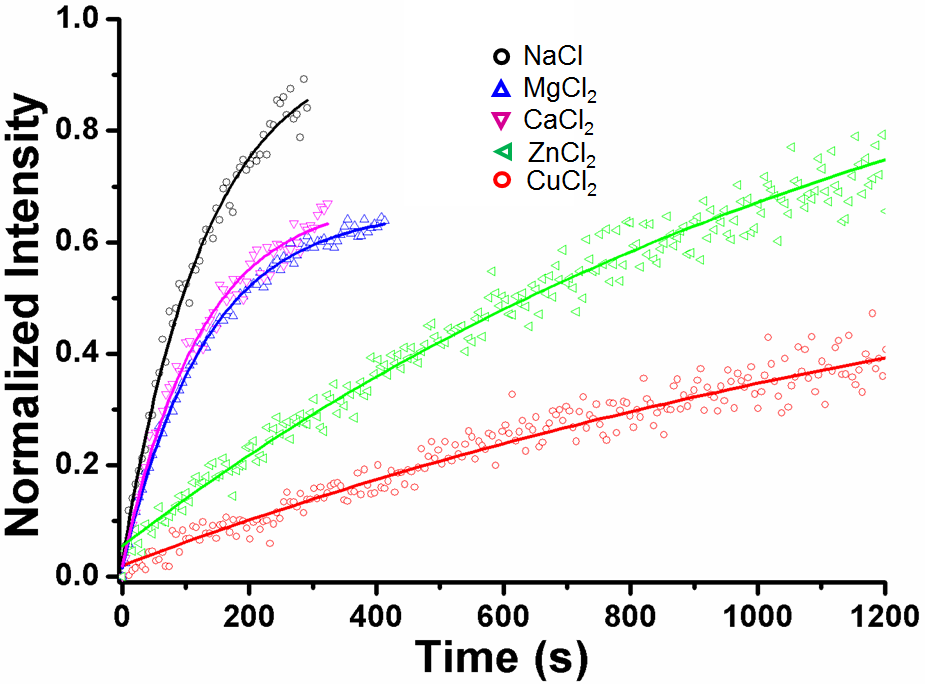


**Supplementary Figure S6.** Fluidity of a DMPC/DMPG bilayer in the presence of metal ions. The normalized fluorescence intensity obtained from the FRAP excitements clear indicate Cu^2+^ has the significant and different effect on membrane fluidity, compared to other ions.

**S7: FRAP experiments on the supported lipid bilayers prepared from DOPG, DOPC and tail labeled NBD-PE** **(1-myristoyl-2-(12-((7-nitro-2-1,3-benzoxadiazol-4-yl) amino)** dodecanoyl)-sn-glycero-3-phosphoethanolamine).


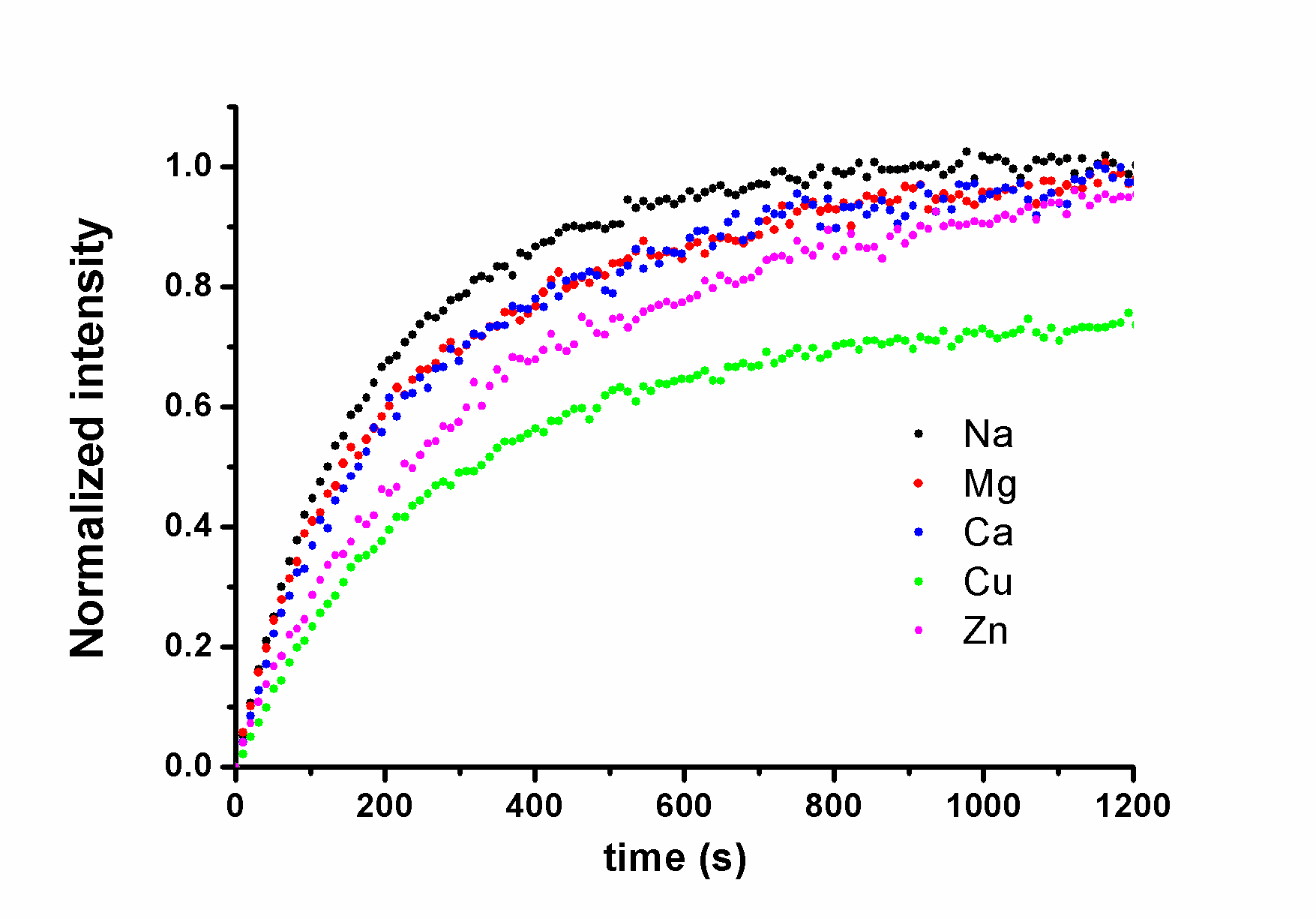

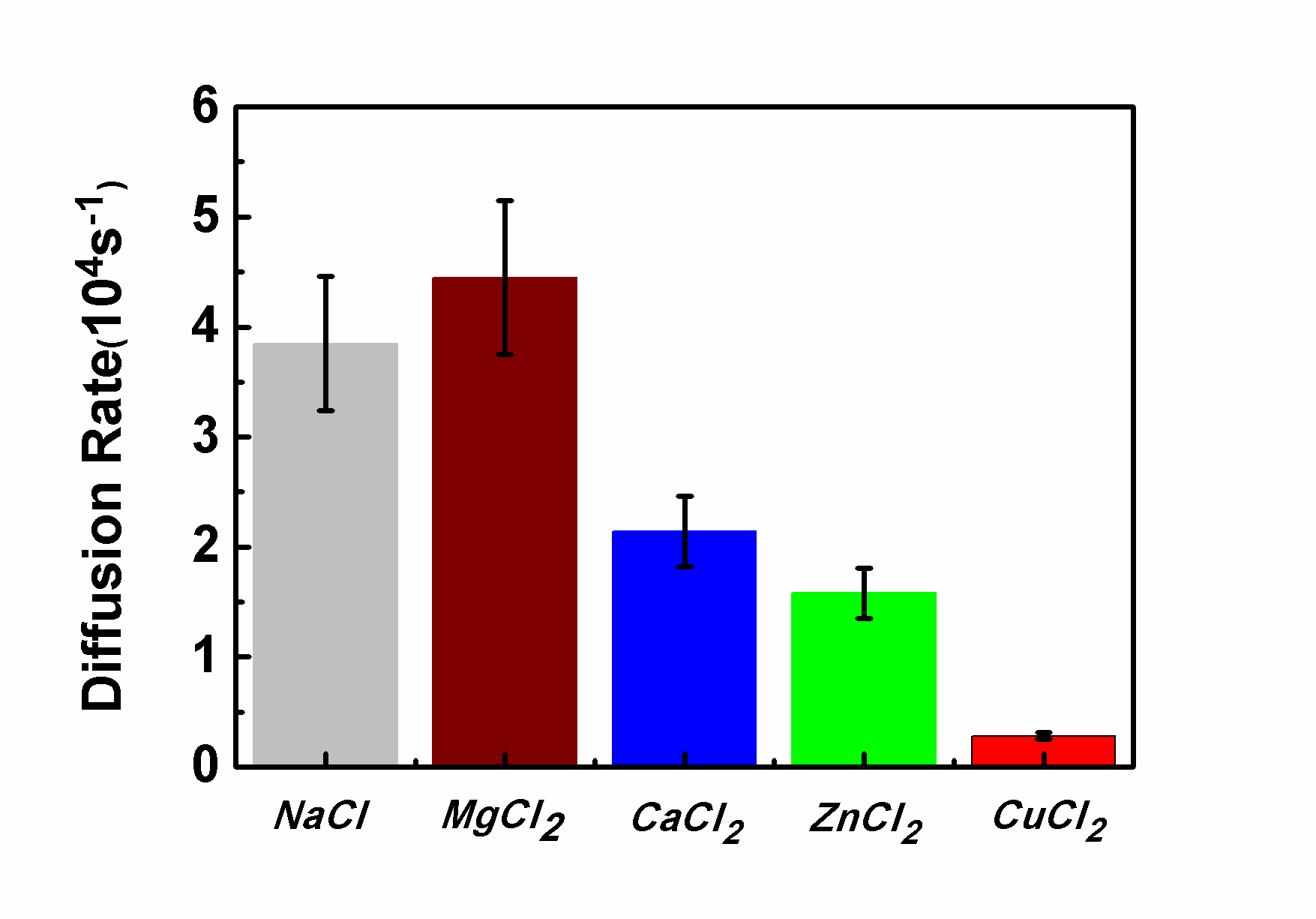


**(a)**

**(b)**

**Supplementary Figure S7.** Fluidity of a lipid bilayer in the presence of metal ions. (a) Normalized fluorescence intensity. The red circle, green triangle, blue inverted triangle, wine diamond and black rectangle indicate the fluorescence data with incubation of Cu^2+^, Zn^2+^, Ca^2+^, Mg^2+^ and control (Na^+^), respectively. The data are fitted with the curves with corresponding colors. The insets are the fluorescence-recovery images with incubation of Cu^2+^ at the times 0 s, 80 s and 160 s. (b) Rates of fluorescence recovery. The rate of incubation with CuCl_2_ is obviously less than those with other buffers, while the rates with MgCl_2_, CaCl_2_ and ZnCl_2_ are comparable.

**Supplementary References**

1. Bader, R. F. W. Everyman's derivation of the theory of atoms in molecules. *J. Phys. Chem. A* **111**, 7966-7972, (2007).

2. Jenkins, S. & Morrison, I. The chemical character of the intermolecular bonds of seven phases of ice as revealed by *ab initio* calculation of electron densities. *Chem. Phys. Lett.* **317**, 97-102 (2000).

3. Espinosa, E., Alkorta, I., Elguero, J. & Molins, E. From weak to strong interactions: a comprehensive analysis of the topological and energetic properties of the electron density distribution involving X–H⋯F–Y systems. *J. Chem. Phys.* **117**, 5529-5542 (2002).

4. Varadwaj, P. R. & Marques, H. M. The physical chemistry of coordinated aqua-, ammine-, and mixed-ligand Co^2+^ complexes: DFT studies on the structure, energetics, and topological properties of the electron density. *Phys. Chem. Chem. Phys.* **12**, 2126-2138 (2010).

5. Dinda, S. & Samuelson, A. G. The nature of bond critical points in dinuclear copper(I) complexes. *Chem. Eur. J.* **18**, 3032-3042 (2012).

6. Jiang, X., Gao, J., Huynh, T., Huai, P., Fan, C., Zhou, R. & Song, B. An improved DNA force field for ssDNA interactions with gold nanoparticles. *J. Chem. Phys.* **140**, 234102 (2014).
